# Measuring quality of life in bariatric surgery: a multicentre study

**DOI:** 10.1007/s00464-019-07350-4

**Published:** 2020-01-28

**Authors:** Youri Q. M. Poelemeijer, Elise T. W. van der Knaap, Perla J. Marang-van de Mheen, Ahmet Demirkiran, Marinus J. Wiezer, Eric J. Hazebroek, Jan Willem M. Greve, Ronald S. L. Liem

**Affiliations:** 1 Scientific Bureau, Dutch Institute for Clinical Auditing, Leiden, The Netherlands; 2 Department of Surgery, Leiden University Medical Center, Albinusdreef 2, 2333 ZA Leiden, The Netherlands; 3 Department of Biomedical Data Science, Leiden University Medical Center, Leiden, The Netherlands; 4 Department of Surgery, Rode Kruis Hospital, Beverwijk, The Netherlands; 5 Department of Surgery, St. Antonius Hospital, Nieuwegein, The Netherlands; 6 Department of Surgery, Rijnstate, Arnhem, The Netherlands; 7 Department of Surgery, Zuyderland Medical Center, Heerlen, The Netherlands; 8 Department of Surgery, Groene Hart Hospital, Gouda, The Netherlands; 9 Dutch Obesity Clinic, The Hague, The Netherlands

**Keywords:** Bariatric surgery, Quality of life, DATO, Netherlands, Roux-en-Y gastric bypass, Sleeve gastrectomy, Proms

## Abstract

**Background:**

Current studies mainly focus on total weight loss and comorbidity reduction. Only a few studies compare Quality of Life (QoL) after sleeve gastrectomy (SG) and Roux-en-Y gastric bypass (RYGB). This study was conducted to examine the extent of improvement in QoL on different domains after primary bariatric surgery and compare these results to Dutch reference values.

**Methods:**

The study included prospectively collected data from patients who underwent primary bariatric surgery in five Dutch hospitals. The RAND-36 questionnaire was used to measure the patient’s QoL; preoperatively and twelve months postoperatively. Postoperative scores were compared to Dutch reference values, standardized for age, using *t*-test. A difference of more than 5% was considered a minimal important difference. A multivariate linear regression analysis was used to compare SG and RYGB on the extent of improvement, adjusted for case-mix factors.

**Results:**

In total, 4864 patients completed both the pre- and postoperative questionnaire. Compared with Dutch reference values, patients postoperatively reported clinically relevant better *physical functioning* (RYGB + 6.8%), *physical role limitations* (SG + 5.6%; RYGB + 6.2%) and *health change* (SG + 77.1%; RYGB + 80.0%), but worse *general health perception* (SG − 22.8%; RYGB − 17.0%). Improvement in QoL was similar between SG and RYGB, except for *physical functioning* (*β* 2.758; *p*-value 0.008) and *general health perception* (*β* 2.607; *p*-value < 0.001) for which RYGB patients improved more.

**Conclusions:**

SG and RYGB patients achieved a better postoperative score in *physical functioning*, *physical role limitations* and *health change compared* to Dutch reference values, and a worse score in *general health perception*.

International literature provides evidence that bariatric surgery can contribute to substantial weight loss and a positive effect on obesity-related comorbidities [1, 2]. On the other hand, bariatric surgery may also lead to severe postoperative complications, as well as endocrine and metabolic complications [1, 3–5]. Psychological consequences of bariatric surgery were also described as complex and not always entirely understood [6–9]. Therefore, bariatric surgery requires a detailed evaluation of its impact on a patients level and is best assessed with a Quality of Life (QoL) assessment [10].

Standard clinical outcomes, such as weight loss and resolution of comorbidities, are mostly objectively measured in registries, resulting in quantitative data, which are convenient for analyses. QoL assessments, however, are primarily patient-reported measurements and may be more challenging to interpret. On the other hand, it is essential to include QoL assessments in the evaluation of health interventions of bariatric surgery as the patient perspective can provide valuable information on the effectiveness of bariatric surgery that cannot be obtained from clinical outcome measures alone [10–12]. QoL could be measured by using questionnaires reflecting the patient’s perspective on the effects of the provided healthcare or treatment given to the patient in their daily lives [10, 11]. Literature showed that the Short Form 36-item Health Survey® (SF-36) is the most commonly used QoL measurement in bariatric surgery [10]. A nearly identical questionnaire is the RAND 36-item Health Survey (RAND-36) that evaluates the same domains as the SF-36. The difference between these two questionnaires mainly consists of the commercial fees required for using the questionnaire [13, 14]. The RAND-36 is also the standard QoL measuring tool offered to patients in all Dutch bariatric hospitals.

Recent studies mainly focussed on clinical outcomes such as total weight loss and obesity-related comorbidity reduction [15, 16]. The few initial studies which compared QoL after sleeve gastrectomy (SG) and Roux-en-Y gastric bypass (RYGB) did not use the RAND-36 and included a low sample size (range 50–1703) with only two studies of more than 1000 patients [17, 18]. Another pitfall in previously conducted studies is the low volume of postoperative respondents as well as single-centre studies, making the results on improvement after bariatric surgery less reliable and also not generalizable to other settings in daily practice [19]. Furthermore, a recent study comparing RYGB and SG in three different European countries showed differences in preoperative characteristics, which may have been the reason for a different surgical approach but could also affect the outcomes including postoperative QoL after bariatric surgery [20]. We therefore compared changes between these procedures, not only looking at statistical significance but also considering clinically relevant differences.

The aim of this study is to compare improvement in QoL after primary bariatric surgery for the two mainly performed primary bariatric procedures in the Netherlands: SG and RYGB. In addition, the study compares postoperative values with reference values for the general Dutch population. A multicentre study design is chosen for a better representation across multiple sites.

## Materials and methods

QoL-data were prospectively collected from all patients undergoing a primary RYGB or SG in the five participating hospitals in the Netherlands between 1 January 2015 and 1 January 2017. QoL-data were linked to the national bariatric DATO-registry covering all centres providing bariatric surgery [21].

The scientific committee, which coordinates the national DATO-registry, represents all participating bariatric centres and all members are mandated by the practising hospital where they practice. This committee approved the research proposal for the present study and manuscript for publication. A more in depth description about the scientific committee is given in an earlier scientific article [21].

### Patients

In the Netherlands, patients with a body mass index (BMI) ≥ 40.0 kg/m^2^ or with a BMI ≥ 35.0 kg/m^2^ and one or more obesity associated comorbidities were eligible for bariatric surgery during the study period [21, 22]. These obesity associated comorbidities were type-2 diabetes mellitus (T2DM), hypertension (HT), dyslipidaemia, gastroesophageal reflux disease (GERD), obstructive sleep apnoea syndrome (OSAS) and musculoskeletal pain. Further treatment strategies, including the choice for SG or RYGB, were determined by a multidisciplinary team and by shared decision making with the patient.

Baseline characteristics in patients undergoing SG or RYGB were compared using the mean ± standard deviation (SD) for normally distributed variables and the median with interquartile range for non-normally distributed variables. The Mann–Whitney *U* test was performed for continuous variables and *χ*^2^ for categorical variables. The threshold for significance has been set at 0.05.

### Quality of life (QoL)

During the development process of the nationwide DATO-registry, several questionnaires were considered including the Bariatric Analysis and Reporting Outcome System (BAROS) [23, 24], SF-36 and RAND-36 questionnaires. Given the controversy surrounding BAROS a generic QoL questionnaire was preferred. The RAND-36 and SF-36 are identical, except for different scoring algorithms for the *pain* and *general health perception* scales, resulting in the choice for the RAND-36 questionnaire [13, 14, 25, 26].

### RAND-36

The Dutch version of the RAND-36 is a validated and standardized translation of the original RAND-36 questionnaire [13, 14]. The questionnaire contains 36 questions within nine scales. These scales are *physical functioning*, *social functioning*, *physical role limitations*, *emotional role limitations*, *mental health*, *vitality*, *pain*, *general health perception* and *health change perception*. Previous studies have shown this to be a valid tool for the measurement of QoL among obese patients undergoing bariatric surgery [25, 27, 28].

Each patient undergoing bariatric surgery in one of the five participating bariatric centres was included in the study. The preoperative questionnaire was completed during the initial screening for bariatric surgery. The postoperative questionnaire was administered 12 months (range 9–15) after primary surgery. The questionnaires were part of the standard given care in the five participating centres.

### Analysing the questionnaire

All completed questionnaires were analysed by a predefined algorithm, provided by the RAND-36 research group and included in the original article [13, 14]. A brief summary is given below.

All scores were recoded following the provided algorithm: a high score indicates a more favourable health state (or outcome) of the patient [14, 26]. Each item was scored on a 0 to 100 range. An average of all scores in each of the nine individual scales has been calculated. Missing values were replaced with the personal mean of the specific scale if at least half of the answers on the questions of the scale were provided [13, 14, 26].

### Comparing to the Dutch reference population

First, postoperative RAND-36 scores were divided into six age groups (18–24, 25–34, 35–44, 45–54, 55–64 and 65 +). Second, the extent of improvement after surgery was calculated by subtracting preoperative RAND-36 scores from the postoperative RAND-36 scores, providing the delta separately for SG and RYGB. Third, the postoperative RAND-36 scores were compared with the Dutch reference values [13], in order to see if patients experience the same QoL postoperatively as the Dutch reference group.

Finally, for a valid comparison, the age distribution of the Dutch reference population [13] was applied to the age-specific QoL-values of the SG or RYGB patients to prevent overall values being different because of a difference in age distribution. The age-standardized QoL scores for each of the nine scales were compared for SG and RYGB patients with the Dutch population using the *t*-test.

### Postoperative influences on the QoL outcomes

The Clavien–Dindo classification (CD) is used to determine whether a patient had experienced a severe postoperative complication [29, 30]. All patients with a CD grade ≥ 3, within 30 days after primary surgery, were registered as severe. Due to the low number of severe complications, both operative techniques have been combined and the T-test compared the severe complicated group with the uncomplicated group.

A distinction has also been made to see whether the achievement of Total Weight Loss (TWL) influences the QoL outcomes [21, 31]. All patients were subdivided into patients who reached 20% TWL within 12 months postoperatively and patients who did not. There were no patients in this cohort missing preoperative or postoperative weight scores. Both groups are compared using the *t*-test (reviewer 2, question 1 and 2).

### Comparison between SG and RYGB

In order to compare between SG and RYB, we compared the extent of improvement between SG and RYGB patients adjusted for patient variables that differed at baseline using multivariate linear regression analysis reporting the beta estimate and *p*-value.

Analyses were performed using R version 3.5.1 and the R-packages ‘Companion to Applied Regression’-package (car 3.0–2), ‘A Grammar of Data Manipulation’-package (dplyr 0.7.8) and ‘Table 1’-package (tableone 0.9.3)’ were used.

**Table 1:** Baseline characteristics

	Sleeve gastrectomy		Roux-en-Y gastric bypass		*p*-value
	*N*	%	*N*	%	
Number of patients	965	19.8%	3899	80.2%	
Gender (female)	745	77.2%	3152	80.8%	0.013*
Weight (median, kg, IQR)	133.9	(119.4–155.0)	123.9	(113.0–136.4)	< 0.001*
BMI (median, kg/m^2^, IQR)	45.8	(42.0–52.7)	43.0	(40.4–46.7)	< 0.001*
Waist circumference (median, cm, IQR)	133	(123–145)	127	(120–136)	< 0.001*
Age (mean, years, SD)	39.3	± 12.5	45.6	± 10.3	< 0.001*
Type 2 diabetes mellitus	170	17.6%	1,160	29.9%	< 0.001*
Hypertension	297	30.8%	1,593	40.9%	< 0.001*
Dyslipidaemia	128	13.3%	902	23.1%	< 0.001*
GERD	49	5.1%	233	6.0%	0.317
OSAS	200	20.7%	812	20.8%	0.965
Musculoskeletal pain	430	44.6%	2,294	58.8%	< 0.001*

Baseline characteristics showing preoperative measurements and prevalence of obesity-related comorbidities

*N* number, *SD* standard deviation, *IQR* interquartile range, *GERD* gastroesophageal reflux disease, *OSAS* obstructive sleep apnoea syndrome

*Statistically significant difference measured

## Results

A total of 5574 unique patients underwent a primary SG or RYGB. Patients who were operated and who did not complete both questionnaires (*n* = 710) were excluded. A total of 4864 (87.3%) patients were eligible for analyses, having completed both a preoperative and postoperative questionnaire. Baseline characteristics were shown in Table 1 and correspond to the national bariatric benchmark in the Netherlands [21].

Patients with a SG were significantly heavier (*p* < 0.001), reflected in both higher BMI and higher waist circumference (*p* < 0.001). Statistically significant differences in obesity-related diseases were seen for T2DM, hypertension, dyslipidaemia and musculoskeletal pain. All these obesity-related diseases were seen significantly more often in the RYGB group (*p* < 0.001) compared to SG.

### Sleeve gastrectomy

For patients undergoing SG, the best postoperative QoL scores were seen in relatively young bariatric patients (Table 2a). However, the older bariatric patients showed a larger positive delta score and therefore showed a bigger improvement compared to the younger and middle-aged patients (Table 2b). For example, the youngest patients scored postoperatively better on *physical functioning* and *physical role limitations*. On the other hand, the delta scores for these domains are slightly lower for the younger patients compared to the oldest group (Table 2a, b).

**Table 2:** Postoperative changes after sleeve gastrectomy

(a)							
Age (*n*)	18–24 (*n* = 135)	25–34 (*n* = 257)	35–44 (*n* = 211)	45–54 (*n* = 226)	55–64 (*n* = 124)	65 + (*n* = 12)	Overall (*n* = 965)
	Mean ± SD	Mean ± SD	Mean ± SD	Mean ± SD	Mean ± SD	Mean ± SD	Mean ± SD
Physical functioning	91.26 ± 13.90	90.42 ± 18.33	88.39 ± 19.81	83.03 ± 20.76	75.27 ± 25.79	58.33 ± 37.77	85.92 ± 20.92
Social functioning	84.68 ± 21.38	86.04 ± 21.47	87.30 ± 19.74	84.03 ± 21.48	82.00 ± 23.28	77.00 ± 34.12	85.03 ± 21.46
Physical role limitations	88.79 ± 26.07	87.57 ± 27.46	85.97 ± 27.93	81.65 ± 32.00	79.30 ± 33.30	70.83 ± 45.87	84.63 ± 29.69
Emotional role limitations	85.37 ± 31.27	86.35 ± 30.38	90.28 ± 26.63	85.49 ± 31.24	83.04 ± 31.02	83.33 ± 40.82	86.42 ± 30.08
Mental health	76.37 ± 17.76	78.21 ± 17.53	81.29 ± 15.41	79.08 ± 18.03	80.99 ± 15.50	72.67 ± 31.94	79.21 ± 17.15
Vitality	61.44 ± 20.74	62.91 ± 18.73	67.90 ± 17.58	67.25 ± 19.69	68.60 ± 18.44	61.67 ± 32.20	65.68 ± 19.20
Pain	82.68 ± 24.76	84.39 ± 21.85	80.48 ± 26.79	75.79 ± 26.33	73.16 ± 27.44	61.00 ± 39.18	79.44 ± 25.72
General health perception	55.68 ± 15.15	56.80 ± 15.96	56.44 ± 15.90	55.42 ± 16.03	51.53 ± 18.00	51.33 ± 24.45	55.48 ± 16.26
Health change	93.68 ± 16.27	93.44 ± 14.83	92.90 ± 17.74	92.49 ± 19.10	93.82 ± 13.11	95.83 ± 10.21	93.18 ± 16.56

(b)							
Age (*n*)	18–24 (*n* = 135)	25–34 (*n* = 257)	35–44 (*n* = 211)	45–54 (*n* = 226)	55–64 (*n* = 124)	65 + (*n* = 12)	Overall (*n* = 965)
	Delta ± SD	Delta ± SD	Delta ± SD	Delta ± SD	Delta ± SD	Delta ± SD	Delta ± SD
Physical functioning	28.51 ± 19.94	30.50 ± 23.68	30.81 ± 26.11	33.61 ± 24.77	33.98 ± 23.26	19.17 ± 42.12	31.46 ± 24.24
Social functioning	8.56 ± 26.69	17.37 ± 29.25	15.64 ± 26.54	14.57 ± 26.20	14.12 ± 25.78	29.33 ± 55.09	14.85 ± 27.48
Physical role limitations	19.54 ± 41.84	28.07 ± 43.76	29.03 ± 42.97	32.66 ± 45.62	27.42 ± 46.63	29.17 ± 62.08	28.28 ± 44.38
Emotional role limitations	0.78 ± 36.75	7.85 ± 40.84	6.45 ± 36.14	10.60 ± 38.88	4.67 ± 42.51	17.00 ± 18.62	6.99 ± 38.94
Mental health	3.17 ± 17.48	10.50 ± 20.20	7.77 ± 17.68	7.68 ± 17.74	6.49 ± 16.02	2.00 ± 28.48	7.65 ± 18.32
Vitality	8.16 ± 19.96	10.61 ± 21.36	14.74 ± 21.50	18.79 ± 20.83	19.09 ± 22.44	0.00 ± 28.28	14.31 ± 21.64
Pain	13.03 ± 25.40	18.66 ± 26.66	18.05 ± 29.37	17.42 ± 26.64	20.12 ± 26.03	26.33 ± 42.07	17.77 ± 27.17
General health perception	18.94 ± 16.61	21.54 ± 17.02	19.38 ± 17.73	19.54 ± 17.42	18.41 ± 17.09	15.33 ± 14.40	19.76 ± 17.20
Health change	54.02 ± 27.72	55.03 ± 29.44	51.94 ± 32.30	53.61 ± 32.98	56.72 ± 30.87	70.83 ± 36.80	54.22 ± 31.02

Postoperative unadjusted scale scores and pre- and postoperative change in scale scores (delta) per age group after *sleeve gastrectomy*, measured with RAND-36

*SD* standard deviation

### Roux-en-Y gastric bypass

For RYGB patients, especially the three youngest categories showed better postoperative scores in almost all RAND-36 domains except for the domain *vitality* and *general health perception* (Table 3a). However, the largest improvement (delta) was seen in the oldest group (65 +). This applies to all domains except *physical role limitations*, *emotional role limitations* and *health change*. For these domains, the second-oldest group (55–64) showed the largest improvement (Table 3b).

**Table 3:** Postoperative changes after Roux-en-Y gastric bypass

(a)							
Age (*n*)	18–24 (*n* = 84)	25–34 (*n* = 537)	35–44 (*n* = 1040)	45–54 (*n* = 1,427)	55–64 (*n* = 741)	65 + (*n* = 70)	Overall (*n* = 3899)
	Mean ± SD	Mean ± SD	Mean ± SD	Mean ± SD	Mean ± SD	Mean ± SD	Mean ± SD
Physical functioning	94.42 ± 10.54	92.90 ± 13.67	91.94 ± 15.58	88.19 ± 18.80	82.17 ± 22.11	84.44 ± 20.23	88.75 ± 18.35
Social functioning	88.42 ± 16.77	83.89 ± 22.03	87.05 ± 21.24	85.07 ± 22.61	84.12 ± 23.07	89.06 ± 20.19	85.40 ± 22.14
Physical role limitations	90.42 ± 24.83	86.17 ± 29.91	87.23 ± 28.79	84.45 ± 31.37	82.22 ± 31.89	87.50 ± 29.44	85.18 ± 30.49
Emotional role limitations	88.82 ± 27.28	83.14 ± 33.50	90.07 ± 26.37	86.61 ± 31.17	87.60 ± 30.28	94.43 ± 20.28	87.44 ± 29.96
Mental health	81.93 ± 12.64	78.02 ± 17.83	81.41 ± 15.96	80.37 ± 17.64	80.36 ± 17.16	85.26 ± 15.90	80.45 ± 17.06
Vitality	62.50 ± 20.14	63.20 ± 19.89	67.19 ± 19.74	67.49 ± 20.30	68.81 ± 19.77	74.54 ± 18.31	67.11 ± 20.04
Pain	87.42 ± 20.75	82.60 ± 23.44	82.76 ± 23.22	79.84 ± 24.59	76.44 ± 25.89	78.56 ± 28.79	80.48 ± 24.45
General health perception	57.47 ± 11.38	57.71 ± 15.01	60.42 ± 14.81	60.14 ± 14.84	59.44 ± 14.79	62.81 ± 15.59	59.75 ± 14.82
Health change	95.42 ± 13.41	94.17 ± 16.92	95.37 ± 15.17	94.61 ± 16.13	94.47 ± 16.59	95.83 ± 15.16	94.77 ± 16.01

(b)							
Age (*n*)	18–24 (*n* = 84)	25–34 (*n* = 537)	35–44 (*n* = 1040)	45–54 (*n* = 1427)	55–64 (*n* = 741)	65 + (*n* = 70)	Overall (*n* = 3899)
	Delta ± SD	Delta ± SD	Delta ± SD	Delta ± SD	Delta ± SD	Delta ± SD	Delta ± SD
Physical functioning	24.17 ± 20.34	30.66 ± 22.87	31.75 ± 23.31	34.54 ± 23.14	36.76 ± 24.36	41.85 ± 24.73	33.61 ± 23.50
Social functioning	8.45 ± 24.03	13.03 ± 26.80	14.20 ± 28.57	13.99 ± 27.51	15.88 ± 28.40	16.13 ± 29.31	14.20 ± 27.84
Physical role limitations	16.25 ± 39.82	23.57 ± 43.65	27.49 ± 44.97	29.19 ± 45.80	33.11 ± 47.96	35.19 ± 47.71	28.57 ± 45.73
Emotional role limitations	4.47 ± 43.26	− 0.97 ± 39.34	6.24 ± 36.86	3.60 ± 40.44	12.83 ± 45.57	9.93 ± 34.10	5.56 ± 40.55
Mental health	7.07 ± 13.77	7.54 ± 18.36	8.44 ± 17.88	6.84 ± 18.46	7.99 ± 18.36	7.26 ± 20.46	7.59 ± 18.23
Vitality	3.75 ± 22.71	12.47 ± 22.74	16.68 ± 23.02	16.98 ± 22.39	18.38 ± 22.89	18.89 ± 22.12	16.32 ± 22.82
Pain	11.58 ± 28.36	17.95 ± 31.18	18.92 ± 27.69	20.99 ± 28.07	20.98 ± 27.36	21.57 ± 33.49	19.85 ± 28.42
General health perception	15.47 ± 18.15	21.40 ± 18.11	22.53 ± 17.34	23.53 ± 17.23	22.89 ± 16.30	23.93 ± 18.13	22.70 ± 17.28
Health change	42.50 ± 30.64	51.49 ± 30.40	55.08 ± 29.28	57.62 ± 28.81	58.16 ± 29.34	57.87 ± 24.68	55.91 ± 29.34

Postoperative unadjusted scale scores & pre- and postoperative change in scale scores (delta) per age group after *Roux-en-Y gastric bypass*, measured with RAND-36

*SD* standard deviation

### Overall quality of life

The comparison of the Dutch reference values with both the postoperative SG and RYGB values were standardized to the age distribution of the Dutch reference group [13]. As suggested in recent scientific literature, a statistical significant QoL-score difference of > 5% is considered a minimal important difference (MID) [32, 33].

Results showed a MID in the domains *physical functioning* (for RYGB), *physical role limitations* and *health change* (for both SG and RYGB) compared to Dutch reference values. Especially, for the domain *health change*, a large difference was observed (Table 4a). However, patients postoperatively still report lower scores on the domain *general health perception*. In addition, the SG scores were slightly lower than the RYGB in all RAND-36 domains (Table 4a).

**Table 4:** Postoperative measured changes compared to the Dutch benchmark

(a)							
	Sleeve gastrectomy			Roux-en-Y gastric bypass			Dutch reference group
	*n* = 965			*n* = 3899			
	Mean ± SD	%	*p*-value	Mean ± SD	%	*p*-value	Mean ± SD
Physical functioning	86.02 ± 19.66	+ 3.5	< 0.001	88.76 ± 17.69	+ 6.8*	< 0.001*	83.09 ± 20.39
Social functioning	85.02 ± 21.32	− 2.7	0.002	85.40 ± 22.05	− 2.2	< 0.001	87.35 ± 19.74
Physical role limitations	84.73 ± 29.21	+ 5.6*	< 0.001*	85.19 ± 30.36	+ 6.2*	< 0.001*	80.24 ± 34.84
Emotional role limitations	86.41 ± 29.90	+ 1.9	0.128	87.43 ± 29.72	+ 3.1	< 0.001	84.79 ± 31.46
Mental health	79.12 ± 17.01	+ 2.8	< 0.001	80.44 ± 16.96	+ 4.5	< 0.001	77.00 ± 18.66
Vitality	65.53 ± 18.98	− 3.0	0.002	67.09 ± 19.92	− 0.7	0.241	67.59 ± 19.82
Pain	79.55 ± 25.15	− 2.0	0.072	80.49 ± 24.27	− 0.8	0.217	81.14 ± 24.37
General health perception	55.50 ± 16.11	− 22.8*	< 0.001*	59.74 ± 14.76	− 17.0*	< 0.001*	71.95 ± 21.60
Health change	93.21 ± 16.29	+ 77.1*	< 0.001*	94.76 ± 15.97	+ 80.0*	< 0.001*	52.63 ± 18.50

(b)							
	Postoperative complication			Unsuccessful %TWL			Dutch reference group
	*n* = 119			*n* = 696			
	Mean ± SD	%	*p*-value**	Mean ± SD	%	*p*-value**	Mean ± SD
Physical functioning	80.58 ± 27.29	− 3.0	< 0.001	82.42 ± 21.50	− 0.8	< 0.001	83.09 ± 20.39
Social functioning	77.90 ± 30.93	− 10.8*	0.003*	81.11 ± 23.50	− 7.4*	0.001*	87.35 ± 19.74
Physical role limitations	75.64 ± 39.88	– 5.7*	0.005*	79.43 ± 33.80	− 1.0	0.002	80.24 ± 34.84
Emotional role limitations	83.71 ± 33.91	− 1.27	0.292	79.16 ± 36.18	− 6.6*	< 0.001*	84.79 ± 31.46
Mental health	78.90 ± 18.81	+ 2.5	0.500	76.38 ± 17.57	+ 0.8	< 0.001	77.00 ± 18.66
Vitality	61.92 ± 22.48	− 8.4*	0.027*	63.47 ± 19.98	− 6.1*	0.004*	67.59 ± 19.82
Pain	70.51 ± 30.66	− 13.1*	< 0.001*	76.72 ± 25.51	− 5.4*	0.015*	81.14 ± 24.37
General health perception	55.74 ± 16.02	− 22.5*	0.060*	53.46 ± 16.37	− 25.7*	< 0.001*	71.95 ± 21.60
Health change	88.46 ± 23.73	+ 68.1*	0.001*	89.72 ± 21.88	+ 70.5*	< 0.001*	52.63 ± 18.50

(a) Represents the postoperative adjusted baseline scale scores for sleeve gastrectomy and Roux-en-Y gastric bypass. (b) represents the postoperative scale scores for patients with a postoperative severe complication (Clavien–Dindo grade ≥ 3) and scale scores for patients with a successful postoperative total weight loss (> 20 %TWL) after 1 year. All outcomes are measured with RAND-36. Percentage difference (%) and *p*-value are comparisons to the Dutch benchmark. *SD* standard deviation, % percentage

*Statistically significant results and clinically relevant difference measured

**p-value measured with the postoperative uncomplicated and the successful %TWL group

### Complications and total weight loss

Hypothetically, a postoperatively complicated course has a negative influence on the postoperative QoL outcomes. To make reliable calculations, both operative techniques were combined and the postoperative complicated group was compared with the uncomplicated group. Table 4b shows that the positive effects in the domains *physical functioning* and *physical role limitations* have disappeared (shown in Table 4a). A MID was seen in the domain of *social functioning*, *physical role limitations*, *emotional role limitations*, *vitality* and *pain*. Patients postoperatively still report lower scores on the domain *general health perception*, while they still report a significant *health change* (Table 4b). (reviewer 2, question 1 and 2).

### SG vs. RYGB

Comparing the extent of improvement between SG and RYGB patients on each domain, a significant difference was seen in the domains *physical functioning* and *general health perception* when adjusted for differences in baseline characteristics; T2DM, hypertension, dyslipidaemia and musculoskeletal pain. These significant differences were mostly seen in the RYGB group (Table 5).

**Table 5:** Postoperative delta scale scores of sleeve gastrectomy and Roux-en-Y gastric bypass

	Sleeve gastrectomy	Roux-en-Y gastric bypass	Beta estimate	*p*-value
	*n* = 965	*n* = 3899		
	Delta mean ± SD	Delta mean ± SD		
Physical functioning	31.46 ± 24.24	33.61 ± 23.50	2.758	0.008*
Social functioning	14.85 ± 27.48	14.20 ± 27.84	− 0.821	0.509
Physical role limitations	28.28 ± 44.38	28.57 ± 45.73	− 1.352	0.505
Emotional role limitations	6.99 ± 38.94	5.56 ± 40.55	− 2.650	0.141
Mental health	7.65 ± 18.32	7.59 ± 18.23	0.483	0.544
Vitality	14.31 ± 21.64	16.32 ± 22.82	0.919	0.361
Pain	17.77 ± 27.17	19.85 ± 28.42	1.148	0.360
General health perception	19.76 ± 17.20	22.70 ± 17.28	2.607	< 0.001*
Health change	54.22 ± 31.02	55.91 ± 29.34	− 0.072	0.957

Postoperative delta scale scores of sleeve gastrectomy and Roux-en-Y gastric bypass, measured with RAND-36. Beta’s were estimated using linear regression and adjusted for T2DM, hypertension, dyslipidaemia and musculoskeletal pain. Compared with a multivariate logistic regression analysis

*SD* standard deviation, *T2DM* type 2 diabetes mellitus

*Statistically significant results and clinically relevant difference measured

## Discussion

Current studies on bariatric surgery particularly focus on weight loss and improvement of obesity-related diseases but do not sufficiently take the patient's perspective into account [21, 34-38]. It is important to focus more on postoperative outcomes from a patient’s perspective, because of the enormous increase in bariatric procedures worldwide [22, 39-41].

There were a few initial studies comparing QoL after SG and RYGB, but these studies had mostly a low sample size and did not use the RAND 36-item Health Survey (RAND-36) [17, 18]. In addition, these studies were almost all single-centre studies and most of them reported low postoperative response rates [19].

However, there were two larger population-based studies comparing postoperative QoL after bariatric surgery. The first study from Waljee et al. had a larger sample size comparing to the previous noted studies, but does not distinguish between SG and RYGB and has a poor follow-up rate [42, 43]. The second study from Sarwer et al. focused on the QoL and sexual functioning of patients with obesity and looked specific on the changes in these domains, but does not made a distinction between RYGB and SG either [44]. As a result, the question remained which postoperative differences in QoL could be measured between the two most commonly used surgery techniques and how these changes relate to the Dutch population. Therefore, the first multicentre study has been conducted comparing QoL between SG and RYGB with a large sample size and a postoperative response rate of more than 85%.

Results showed that bariatric patients had meaningful higher postoperative scores on *physical functioning*, *physical role limitations* and *health change* for both SG and RYGB compared to Dutch reference values, but meaningful lower scores on *general health perception*. It may be concluded that patients feel better postoperatively, but not yet fully healthy (reviewer 1, question 3). These results could be a prelude to focus more on these domains, so that bariatric patients do not end up in social isolation and feeling healthier, similar to the national average.

Table 4b clearly showed that a postoperatively severe complicated course or failure to achieve the desired weight loss influences the QoL outcomes. Where first a meaningful positive postoperative score in *physical functioning* and *physical role limitations* was seen (Table 4a), a significant negative score was now seen in the severe complicated group and the unsuccessful %TWL group. In addition, a negative trend is also observed in almost all other domains. This argues for better psychological postoperative support for patients where the outcomes do not meet Textbook Outcome [45] (reviewer 2, question 1 and 2).

The two bariatric surgical techniques showed a similar QoL improvement in all domains except for *physical functioning* and *general health perception* for which RYGB patients showed a higher postoperative improvement. This difference could be explained by the underlying indication for treatment. Particularly for patients with a high BMI (> 50 kg/m^2^) a SG is preferably, so a second-stage procedure may follow [46]. The use of the SG for morbid obese patient stems from the use of this procedure as a modification to the duodenal switch. Later on, it was used as a first part of a two-stage gastric bypass procedure on morbid obese patients. In the beginning of this century multiple studies have been published with a laparoscopic sleeve gastrectomy as an isolated bariatric procedure, with promising results [47, 48]. During the time when this study was conducted, the RYGB was often used as a second-stage procedure in Dutch bariatric hospitals. In recent years and increase in the use of the "one anastomosis gastric bypass" (OAGB) and the "single anastomosis duodeno-ileal bypass with sleeve gastrectomy" (SADI-s) was seen. In addition, minor modifications have been applied to the existing RYGB. This makes the RYGB more successful in patients with a higher BMI. This has led to a decrease in the number of SG procedures nowadays (reviewer 1, question 1 and 2).

Patient with a SG could experience a worse health perception compared to patients with a single RYGB operation. Despite the fact that BMI is added in the case-mix model, also the weight loss in both groups can be experienced differently.

Another significant difference was seen in the preoperative registered obesity-related diseases in the RYGB group. Several studies suggest that the RYGB has a greater beneficial effect on obesity-related diseases after surgery compared to SG [49–52]. This also could have an effect on the surgeon's choice for the type of bariatric surgery and therefore the differences in experienced QoL.

But the proper interpretation of these results remains a point of discussion. As has been shown for other type of surgeries and diseases, the RAND-36 is a generic questionnaire and may not be specific enough to fully analyse the QoL in bariatric patients [53, 54]. For example, when looking at *physical functioning*, the score was calculated on the basis of ten questions. These questions relate to typical activities during the day and may be one of the key items for patients with obesity, but can only be answered with a limited number of options; limited a lot, limited a little or not limited at all which is likely to capture only the very severe physical limitations e.g. induced by severe obesity. More subtle differences may not be measured adequately, while this is essential for obese and bariatric patients. Using a bariatric-specific quality of life questionnaire may detect more clinically relevant differences. However, as already mentioned in the introduction, there were currently no suitable bariatric questionnaires that could be applied in current scientific research.

Another point of debate is calculating and reporting the MID. Not only the baseline scores may vary by population and context, but also the differences experienced and noticed by the patient may vary. Therefore the proper interpretation of these results remains a point of discussion [55]. This means, for example, that an increase of 10 points should be interpreted differently if the baseline values differ. And also, an individual rise from 10 to 20 on a 100-point scale can be interpreted differently than a rise from 60 to 70.

As mentioned earlier in the discussion, other studies have not shown differences between SG and RYGB in QoL, while different outcomes following these two operations can be hypothesized [17, 18]. For example, the indication for the type of bariatric surgery is mainly based on the choice and expertise of the specific surgeon. There were some studies that suggest that the RYGB has a more beneficial effect on metabolic obesity-related diseases [46]. We tried to correct this by adding these baseline differences (Table 1) in the case-mix model.

There are some limitations, despite the accuracy of this study. A possible response bias could be generated by excluding patients from the study without postoperative measurements. However, this study offers a high response rate, whereby it can be assumed that the influence will be very small. In addition, this study didn't focus on statistically significant differences alone but also described whether these differences were clinically relevant (minimal important difference; MID) which at the same time will also safeguard against finding chance differences. This study does not have specific data to make a comparison between patients who participate in a multidisciplinary postoperative coaching programme and patients who did not. These coaching programmes could consist of participating in support groups, appointments with a dietician and psychological follow-up by trained professionals (reviewer 2, question 3).

There is still no consensus, whether to correct for multiple testing or not. Current statements say, an adjustment is particularly required in confirmatory analyses with multiple analyses stating one final conclusion [56]. This study, on the other hand, has an exploratory meaning with the aim of not missing potentially important findings by a standard adjustment of multiple testing [57]. Therefore, the results in this study were not corrected for multiple testing.

The strength of this study was the large sample size, high response rate and the prospective study design. Most other studies collecting patient-reported outcome data struggle to get a sufficiently high response rate as a part of daily routine clinical care. Therefore, the number of requested items has been kept to a minimum, but at the cost of having limited other data to e.g. adjust for patient characteristics. Furthermore, this was a multicentre study including hospitals located in different geographic areas; therefore, a representative group from the population was obtained. A limitation of the current study was the availability of only one-year follow-up data. When all Dutch hospitals have implemented the PROMs registration, the follow-up will be extended to an annual follow-up up to five years after the primary surgery in the national DATO-registry. This will further substantiate the current outcomes of this study.

## Conclusion

This study showed that bariatric patients achieve better postoperative *physical functioning*, *physical role limitations* and *health change* for both SG and RYGB compared to Dutch reference values, but worse general health perception. In addition, a larger improvement in *general health perception* was seen in patients who underwent RYGB compared to SG. Further studies are needed to develop a specific QoL questionnaire, which focuses on the different aspects of the bariatric patient and the different inclusion criteria for a specific procedure.
